# *Enterocytozoon Bieneusi* Infects Children With Inflammatory Bowel Disease Undergoing Immunosuppressive Treatment

**DOI:** 10.3389/fmed.2021.741751

**Published:** 2021-09-30

**Authors:** Żaneta Zajączkowska, Katarzyna Akutko, Martin Kváč, Bohumil Sak, Magdalena Szydłowicz, Andrzej B. Hendrich, Barbara Iwańczak, Marta Kicia

**Affiliations:** ^1^Department of Biology and Medical Parasitology, Wrocław Medical University, Wrocław, Poland; ^2^Department and Clinic of Paediatrics, Gastroenterology and Nutrition, Wrocław Medical University, Wrocław, Poland; ^3^Biology Centre of the Czech Academy of Sciences, Institute of Parasitology, Ceské Budějovice, Czechia; ^4^Faculty of Agriculture, University of South Bohemia, Ceské Budějovice, Czechia

**Keywords:** *Enterocytozoon bieneusi*, inflammatory bowel disease, children, immunosuppressive treatment, molecular characterization

## Abstract

**Objectives:** Patients with inflammatory bowel disease (IBD) are susceptible to intestinal opportunistic infections due to both defective mucosal immunity and altered immune response resulting from immunosuppressive treatment. Microsporidia infecting the gastrointestinal tract and causing diarrhoea can potentially affect the course of IBD.

**Methods:** Stool samples (90 IBD children and 121 healthy age-matched controls) were screened for *Encephalitozoon* spp. and *Enterocytozoon bieneusi* by microscopy and polymerase chain reaction followed by sequencing.

**Results:** *E. bieneusi* genotype D was found in seven out of 90 (7.8%) IBD children. No children from the control group were infected, making the pathogen prevalence in the IBD group significant (*P* = 0.002). Furthermore, infection was confirmed only in patients receiving immunosuppressive treatment (*P* = 0.013).

**Conclusions:** Children with IBD are at risk of intestinal *E. bieneusi* infection, especially when receiving immunosuppressive treatment. Therefore, microsporidia should be considered as a significant infectious agent in this group of patients.

## Introduction

Inflammatory bowel disease (IBD) is a complex, chronic inflammatory condition of the gastrointestinal tract developing as a result of the interaction of genetic and environmental factors. The two main types of IBD are Crohn's disease (CD) and ulcerative colitis (UC). Although the aetiology of IBD is still poorly understood, the role of the gut microbiota combined with genetic susceptibility in disease pathogenesis is highlighted (1). Since immunomodulators are commonly used in the treatment of IBD, patients' immune response is compromised making them more susceptible to opportunistic infections (2). Currently, IBD has evolved into a worldwide disorder with rising prevalence in paediatric populations and age is an additional, independent risk factor for opportunistic infections (3). To date, the majority of reported infections among IBD patients have been caused by bacteria and viruses (4). Reports on fungal infections in the course of IBD are comparatively rare (5, 6).

*Enterocytozoon bieneusi* and species from the genus *Encephalitozoon* (*E. intestinalis, E. cuniculi* and *E. hellem*) are the most clinically significant microsporidia – opportunistic fungi infecting eukaryotic cells (7). Due to a wide range of animal hosts, microsporidial spores are distributed in the environment, contaminating water and food, and infecting hosts through faecal-oral or inhalation routes (8, 9). Since their spores initially develop in the epithelium of the small intestine, diarrhoea, abdominal pain, fever, nausea, malabsorption and weight loss are the most common symptoms of microsporidiosis (10). As opportunistic pathogen, microsporidia cause more severe and even fatal infection mainly in immunosuppressed patients, such as HIV-infected or organ and bone marrow transplant recipients (11, 12). In turn, in immunocompetent people such an infection usually manifests as a self-limiting diarrhoea or stays asymptomatic. Such a latent infection may, however, affect the course and symptoms of basic disease entity in patients with chronic disorders (13).

Here, we present the prevalence of microsporidia in the gastrointestinal tract of children with IBD, both with CD and UC. The role of microsporidial infection in the course of IBD has not been well-studied so far and available literature is restricted to adult patients only. To the best of our knowledge, this is the first report concerning microsporidial infection among IBD children.

## Materials and Methods

### Patients

Samples from two groups of HIV-negative patients under the age of 18 years were analysed between 2014 and 2017: (i) children with IBD (study group; *n* = 90), including children with CD (57/90) and UC (33/90), who had been under the care of the 2nd Department and Clinic of Paediatrics, Gastroenterology and Nutrition (Wroclaw Medical University, Poland) and (ii) healthy children (control group; *n* = 121) matched by age with the study group. The revised Porto Criteria recommended by the European Society for Paediatric Gastroenterology, Hepatology and Nutrition (ESPGHAN) were used for the diagnosis of IBD (14). Clinical CD and UC activity were assessed according to the Paediatric Crohn's Disease Activity Index (PCDAI) and the Paediatric Ulcerative Colitis Activity Index (PUCAI), respectively. IBD patients were divided according to three clinical frameworks: (i) 43 (47.8%) patients with active disease presenting at, or shortly after, diagnosis with no previous treatment for IBD, (ii) 20 (22.2%) patients with remission (PCDAI/PUCAI scores <10 for at least 12 months) and (iii) 27 (30%) patients with active disease (mild: PCDAI/PUCAI scores 11–25; moderate: PCDAI/PUCAI scores 26–50; severe: PCDAI/PUCAI scores >51 and symptoms of disease).

The immunosuppressive treatment was administered in 52.2% (47/90) of all IBD children, according to current clinical practise. Four groups of immunosuppressive drugs: (i) glucocorticoids, (ii) azathioprine, (iii) methotrexate and (iv) infliximab or adalimumab were implemented in various configurations as shown in Table 1. Of 47 patients with immunomodulatory treatment, 38 were treated with one drug only (80.9%), eight with two drugs (17%) and one with three drugs (2.1%). Moreover, at the time of the study, 16.7% (15/90) of IBD children were being treated with metronidazole. Adequate antibiotics were administered in case of five children diagnosed with bacterial infection.

**Table 1:** Comparison of *Enterocytozoon bieneusi*-positive and -negative IBD children's basic characteristics.

**Characteristic**	***Enterocytozoon bieneusi***		***P*** **-value**
	**Positive (*N* = 7)**	**Negative (*N* = 83)**	
**Age**			
Median years (range)	11 (10-17)	14.5 (6-17)	0.117
**Sex**			
Male	3 (42.9)	48 (57.8)	0.461
Female	4 (57.1)	35 (42.2)	0.461
**Type of IBD**			
CD	5 (71.4)	52 (62.7)	1.000
UC	2 (22.2)	31 (38.3)	1.000
**Symptoms**			
Fever	0	2 (2.4)	1.000
Diarrhoea	2 (28.6)	28 (33.7)	1.000
**Microbiological diagnosis**			
*Yersinia*	0	1 (2.4)	1.000
*Salmonella* group D	0	1 (2.4)	1.000
*Clostridium difficile* toxins	0	3 (7.1)	1.000
**Metronidazole**	2 (28.6)	13 (15.7)	0.330
**Immunosuppressive regimen**			
general immunosuppression	7 (100)	40 (48.2)	0.013^*^
glucocorticoids monotherapy	0	5 (6.0)	1.000
azathioprine monotherapy	6 (85.7)	22 (26.5)	0.003^*^
adalimumab/infliximab monotherapy	0	5 (6.0)	1.000
azathioprine + glucocorticoids	1 (14.3)	7 (8.4)	0.491
glucocorticoids + methotrexate + infliximab	0	1 (1.2)	1.000

*Data represent number (%) unless otherwise indicated; CD, Crohn's disease; UC, ulcerative colitis;*

* *P-value < 0.05*.

Apart from the diagnosis of IBD, there were no other criteria for participation in the study group. Among clinical data, diarrhoea (defined as at least 3 watery bowel movements per day) and fever (body temperature above 38°C) at the time of examination were taken into account. Additionally, standard laboratory microbiological examinations were carried out among IBD children to identify infections with bacteria of the genus *Salmonella, Shigella, Campylobacter, Yersinia* and toxins A and B of *Clostridium difficile* during acute exacerbation of the basic disease. The following exclusion criteria have been applied for control group: (i) acute infections, (ii) inflammatory, autoimmune or immunodeficiency diseases, and (iii) no previous immunosuppressive treatment history of note.

### Sample Collection

Single stool samples were collected into disposable sterile containers from all of the patients. In the case of three IBD children additional sampling after implementation of immunosuppressive treatment was performed. Each sample was split into two parts, one part was immediately fixed in high quality methanol for microscopy and second part was stored, without preservatives at −20°C for 1–2 weeks before molecular analyses.

### Microscopic Examination

Standard Calcofluor M2R staining method was used to detect microsporidial spores (15). Briefly, dry methanol-fixed smears were stained with 0.1% Calcofluor M2R (Sigma-Aldrich, St. Louis, MO, USA) in phosphate-buffered saline (PBS) for 10 min, rinsed gently with PBS, stained with 0.5% Evans blue (Sigma-Aldrich, St. Louis, MO, USA) in distilled water for 30 s, rinsed again in PBS, and allowed to air dry. Stained slides were examined by fluorescence microscopy using UV light and at a magnification of 1,000x.

### DNA Isolation

For DNA extraction, a total of 200 mg of stool was initially homogenised by bead disruption using a Precellys 24 Instrument (Bertin Technologies, France). Genomic DNA (gDNA) was extracted using the GeneMATRIX Stool DNA Purification Kit (EurX, Gdańsk, Poland) according to the manufacturers' instructions and stored at −20°C for molecular analyses.

### Molecular Examination

gDNA was analysed by genus-specific nested polymerase chain reaction (nested-PCR) protocols amplifying a partial sequence of a small subunit of ribosomal RNA gene (16S rRNA), the entire ITS (internal transcribed spacer) region, and a partial sequence of the 5.8S rRNA gene of *E. bieneusi* and *Encephalitozoon* spp (16–18). Sequences of primers used in primary and secondary nested-PCR are shown in Table 2. Negative (molecular grade water) and positive controls (DNA extracted from *E. bieneusi* genotype CZ3, *E. cuniculi* genotype III spores) were included in each PCR amplification. All manipulations during DNA extractions and amplifications were performed in separate areas of the laboratory, in laminar flow cabinets, previously decontaminated by UV light and disinfectants with international aseptic attestations. Disposable pipettes, tubes and reagent aliquots were used to avoid contamination.

**Table 2:** Sequences of oligonucleotides used for specific *Enterocytozoon bieneusi* and *Encephalitozoon* spp. rDNA amplification and direct sequencing.

**Species**	**Reaction step**	**Primer**	**Sequence (5'- 3')**	**Final amplicon (bp)**
*Enterocytozoon bieneusi*	Primary	EBITS3	GGTCATAGGGATGAAGAG	
		EBITS4	TTCGAGTTCTTTCGCGCTC	
	Secondary	EBITS1*	GCTCTGAATATCTATGGCT	~390 – depending on genotype
		EBITS2_4^*^	ATCGCCGACGGATCCAAGTG	
*Encephalitozoon* spp.	Primary	INT580F	TGCAGTTAAAATGTCCGTAGT	
		INT580R	TTTCACTCGCCGCTACTCAG	
	Secondary	MSP_3^*^	GGAATTCACACCGCCCGTCvyTAT	*E. cuniculi* (~305), *E. intestinalis* (289)
		MSP_4A^*^	CCAAGCTTATGCTTAAGTymAArGGGT	*E. hellem* (~315)

* *oligonucleotides used for direct sequencing*.

### Sequencing and Phylogenetic Analyses

Secondary PCR products were sequenced in both directions using the Sanger sequencing method. Amplification and sequencing of each sample were repeated twice. Nucleotide sequences were edited using the program ChromasPro 2.1.5 and aligned with each other and with reference sequences from GenBank (www.ncbi.nlm.nih.gov/blast) using MAFFT version 7 (http://mafft.cbrc.jp/alignment/software/). Phylogenetic analyses were performed using MEGA7 software and trees were inferred by the maximum likelihood (ML) method. Bootstrap support for branching was based on 1,000 pseudoreplicates.

### Statistical Analysis

The Fisher's exact test was used to compare categorical variables (sex, type and activity of IBD, presence of fever and/or diarrhoea, applied immunosuppressive treatment and metronidazole) between microsporidia-positive and -negative patients, while the continuous variable (age) was compared using Student's *t*-test. A value of *P* < 0.05 was considered significant.

### Ethics Approval Statement

The study was approved by the Human Research Ethics Committee of Wrocław Medical University (KB-24/2014). Written consent was provided by the parents or legal guardians on behalf of all the paediatric patients involved in the study.

## Results

Among all IBD children (*n* = 90) the median age of patients was 14 years, and ranged between six and 17 years, while in children of the control group (*n* = 121) the median age was 10 years, and ranged between three and 16 years. Overall, the male-to-female ratio was 51 (56.7%) to 39 (43.3%) in the study, and 58 (47.9%) to 63 (52.1%) in the control group.

At the time of examination, diarrhoea and fever were observed in 30 and two IBD patients, respectively. *Yersinia* and *Salmonella* group D infections were confirmed in one child each, while *Clostridium difficile* toxins were identified in three children among 42 subjected to microbiological examination.

**Figure 1:**
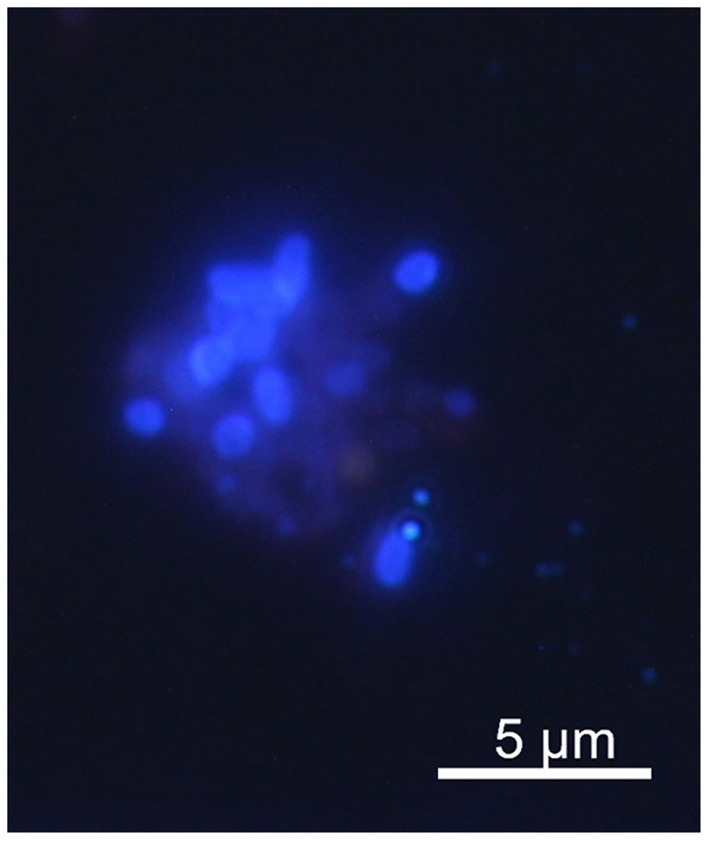
Microsporidial spores identified in Calcofluor M2R staining of IBD patient stool.

None of the children tested were positive for the presence of specific DNA of *Encephalitozoon* spp. Out of 90 IBD children, seven (7.8%) were PCR positive for *E. bieneusi* (Table 1). No infection caused by these microsporidia was confirmed among children from the control group, making the *E. bieneusi* prevalence in IBD patients statistically significant (*P* = 0.002; data not shown). Phylogenetic analysis revealed the presence of *E. bieneusi* genotype D in all samples. Spores were confirmed microscopically in five out of seven (71.4%) samples (Figure 1). No co-infection with other screened bacteria was observed in any *E. bieneusi-*positive patient.

The *E. bieneusi* infection was confirmed exclusively in a group of children receiving immunosuppressive treatment, which is 14.9% (7/47) of the patients in that group (*P* = 0.013), regardless of its duration (1.5–43 months). Six out of seven (85.7%) *E. bieneusi*-positive IBD patients received azathioprine as a single drug (*P* = 0.003), while one patient received simultaneous therapy with azathioprine and glucocorticoids. Out of three patients diagnosed before and after azathioprine implementation, two were positive for *E. bieneusi*, and infection was confirmed only after immunosuppressant treatment. *E. bieneusi* infection was observed in patients with various activity of IBD (Table 3). No significant associations were found for the other tested variables (*P* > 0.05; Table 1).

**Table 3:** Clinical and demographic characteristics of *Enterocytozoon bieneusi*-positive children at the time of examination.

**Case No**.	**Detection of *E. bieneusi***	**Sex**	**Age (years)**	**Inflammatory bowel diseases**			**Activity of IBD**	**Immunosuppressive treatment**		**Other treatment**	**Presence of diarrhoea**
				**Type**	**Localisation**	**Duration (years)**		**Type**	**Duration (months)**		
9*	MIC/PCR	female	11	UC	Colon	8.0	mild	azathioprine	25.0	-	+
33	MIC/PCR	female	12	CD	stomach, colon	5.0	mild	azathioprine	23.0	-	-
51	PCR	female	11	CD	stomach, ileum, colon	0.5	mild	azathioprine	4.0	metronidazole	-
66*	MIC/PCR	male	17	CD	stomach, duodenum, colon	1.0	moderate	azathioprine	11.0	metronidazole	-
82	PCR	female	16	UC	colon	4.0	remission	azathioprine	43.0	-	-
85	MIC/PCR	male	11	CD	Colon	4.0	remission	azathioprine, glucocorticoids	1.5	-	+
89	MIC/PCR	male	10	CD	Colon	2.5	remission	azathioprine	29.0	-	-

* *patients examined before and after implementation of immunosuppressants; CD, Crohn's disease; UC, ulcerative colitis*.

Detailed clinical and demographic characteristics of *E. bieneusi*-positive patients are shown in Table 3.

## Discussion

Routine examination of infectious agents among IBD individuals includes mainly bacteria and viruses. In our study we found *E. bieneusi*—intracellular parasitic fungus—in 7.8% of children with IBD, while none of the patients from the control group was infected, and this difference was statistically significant. Similar results have been shown in stool samples collected from adults with IBD receiving immunosuppressive agents, where 12.7% of patients were infected with *E. bieneusi* (19). One of the reasons for increased susceptibility of IBD patients to such opportunistic infections and/or the reactivation of latent infection might be the immune deterioration as a result of immunosuppressive treatment implemented to achieve IBD remission and/or to prevent disease flares. Indeed, in our study *E. bieneusi* was confirmed exclusively in children with IBD receiving immunosuppressive treatment. Since the duration of immunosuppressive therapy in *E. bieneusi*-positive patients varied from 1.5 to 43 months, even temporary immunomodulatory treatment may be a risk factor for *E. bieneusi* infections. This may be confirmed by the detection of *E. bieneusi* infection in two of our patients after, but not before treatment with immunosuppressive drug, azathioprine.

On the other hand, the ongoing inflammatory process in the course of IBD might facilitate inhabitation of the intestine by pathogens. Since IBD affects the gastrointestinal tract, the influence of this disorder on increased susceptibility to infection may result from the degradation of the intestine and defective mucosal immunity (5). In the course of CD as well as of UC inflammatory infiltrates appear within the intestinal mucosa, destroying mucosal and, in case of UC, also submucosal membrane. Moreover, mucosal inflammation as a result of alterations in the structure and/or quantity of mucins and claudins, could make the intestine more vulnerable to penetration of pathogen (20, 21). It would allow the pathogen to persist in the intestinal mucosa, making the process chronic (5). Furthermore, microsporidia could persist and replicate inside macrophages and in this form, they can be transported throughout the host (22). Brdíčková et al. (23) have shown that other microsporidial species, *E. cuniculi* genotype II, migrates to inflammatory foci probably with the contribution of immune cells participating in its formation (23). If this is the case in patients suffering from chronic inflammatory process, subsequent worsening of the IBD clinical picture might occur.

Epidemiological studies have shown that microsporidiosis among individuals with an impaired immune system is symptomatic, including primarily diarrhoea and fever (12). Since diarrhoea might be a symptom of IBD as well, the difficulties in differentiation the causative agent in cases of infection is generally highlighted (24). Nevertheless, only a minority of microsporidia-positive samples were diarrheal and no significant association between infection and diarrhoea or fever was observed in patients in this study. However, as microsporidia can persist as a latent infection (25), there is a risk of development of symptoms, for instance as a result of increasing the immunosuppressive treatment dose. Moreover, infection with intracellular pathogens in patients with chronic inflammatory process may cause deepening in tissue damage in the place where inflammation persists and worsening of the clinical picture of the disease. Therefore, the need for diagnosing of such infections, especially in immunosuppressed patients, even asymptomatic, is emphasised by the risk of development of more severe symptoms, their incomprehensible impact on the course of the disease and the complexity or the effectiveness of the therapy process.

Interestingly, all of our patients were infected with *E. bieneusi*, similarly as in the report of Hasani et al. (19). Genotype D which is widespread among different groups of patients, both immunosuppressed an immunocompetent (12) and has a broad geographic range (26), was identified in all these samples. However, regardless of the high *E. bieneusi* prevalence among immunocompetent people (25), intestinal microsporidial infections, especially in immunosuppressed patients, can be associated also with *Encephalitozoon* species, mainly *E. intestinalis* and *E. cuniculi*. There are studies suggesting that metronidazole, drug used in IBD therapy, might be able to reduce the number of cells infected with *Encephalitozoon* species *in vitro* (27) and *E. bieneusi* responds only temporarily to treatment with metronidazole (28). However, according to the study by Lallo et al. (29) metronidazole is not effective against microsporidia. A similar conclusion can be drawn from clinical studies (30). Therefore, it is difficult to conclude if metronidazole treatment applied in 16.7% of IBD children had an influence on the microsporidial species identified in this study. Moreover, the question as to whether or not processes taking place in the intestine of IBD patients promote in some way inhabitation selectively by *E. bieneusi* remains unknown and requires detailed investigation. Nevertheless, detailed characterisation of microsporidial species is essential due to the fact that microsporidia treatment is limited and genus-specific. Albendazole is the drug of choice for *Encephalitozoon* spp. treatment and even though it is relatively well-tolerated, this drug has limited efficacy against *E. bieneusi*, especially in patients with impaired immune systems (30). Fumagillin reveals higher efficacy against *E. bieneusi* but shows toxicity and may not be fully effective in eradicating the pathogen (31). In such cases, rehabilitation of the immune system by dose reduction or temporary withdrawal of the immunosuppressive drug may be beneficial and lead to the pathogen elimination without the need for specific treatment (32).

In conclusion we have shown that children with IBD should be considered as a group at risk of microsporidial intestinal infections, especially when receiving immunomodulatory treatment. Even though we have not found any correlation between *E. bieneusi* infection and course of illness, the impact of such intestinal infection on the inflammatory processes in the bowel of IBD patients cannot be excluded. Since most of *E. bieneusi*-infected patients were asymptomatic such an unnoticed infection might develop to a chronic condition, as a result leading to progressive intestine devastation. Therefore, it seems that microsporidia should be considered during diagnosis of infectious agents affecting children suffering from IBD.

## Data Availability Statement

The raw data supporting the conclusions of this article will be made available by the authors, without undue reservation.

## Ethics Statement

The study was approved by the Human Research Ethics Committee of Wrocław Medical University (KB-24/2014). Written informed consent to participate in this study was provided by the participants' legal guardian/next of kin.

## Author Contributions

ŻZ and MKi designed research. ŻZ, KA, MK, BS, MS, and MKi performed research and analysed data. ŻZ, KA, and MKi wrote the manuscript and MK, BS, MS, AH, and BI revised of the manuscript for important intellectual content. All authors had full access to all the data in the study and had final responsibility for the decision to submit the manuscript for publication.

## Funding

This work was supported by the Ministry of Health subvention (grant numbers STM.A060.20.093 and STM.A060.20.105) from the IT Simple system of Wroclaw Medical University and the Czech Science Foundation (GACR 20-10706S). The funders had no role in study design, data collection and analysis, decision to publish, or preparation of the manuscript.

## Conflict of Interest

The authors declare that the research was conducted in the absence of any commercial or financial relationships that could be construed as a potential conflict of interest.

## Publisher's Note

All claims expressed in this article are solely those of the authors and do not necessarily represent those of their affiliated organizations, or those of the publisher, the editors and the reviewers. Any product that may be evaluated in this article, or claim that may be made by its manufacturer, is not guaranteed or endorsed by the publisher.
